# Social withdrawal and neurocognitive correlates in schizophrenia

**DOI:** 10.1097/YIC.0000000000000395

**Published:** 2022-01-31

**Authors:** Domenico De Donatis, Stefano Porcelli, Diana De Ronchi, Emilio Merlo Pich, Martien J. Kas, Amy Bilderbeck, Alessandro Serretti

**Affiliations:** a Department of Biomedical and NeuroMotor Sciences, University of Bologna, Bologna, Italy; b Research & Development, Alfasigma Schweiz, Zofingen, Switzerland; c Groningen Institute for Evolutionary Life Sciences, University of Groningen, Groningen, the Netherlands; d P1vital Ltd, Wallingford, UK

**Keywords:** neurocognition, schizophrenia, social withdrawal

## Abstract

Poor neurocognitive performance has been associated with poor functional outcome in schizophrenia (SCZ) in past studies. Nonetheless, the likely association between neurocognition and social withdrawal has never been investigated. The aim of our study was to investigate in a large and heterogeneous sample of SCZ patient cross-sectional associations between neurocognitive domains and social withdrawal. The sample included 761 SCZ patients who completed the baseline visit in the CATIE study. Neurocognition was assessed by a comprehensive battery of tests resulting in five domain scores and a composite score. Social withdrawal was measured by a specific item of the Heinrichs-Carpenter Quality of Life Scale. Social withdrawal was associated with a lower score in the neurocognitive composite score and in ‘Verbal memory,’ ‘Processing speed’ and ‘Working memory’ scores. ‘Verbal memory’ score showed the strongest association with social withdrawal. Eight percent of the total variance of social withdrawal was explained by these three cognitive domains and additional clinical and sociodemographic factors (education years, PANSS positive symptoms score, and employment). Our results confirmed the wide heterogeneity and specificity of the correlation between neurocognitive domains and indicators of functional outcome in SCZ, underlining the role of certain neurocognitive abilities in social withdrawal.

## Background

One of the most relevant target of treatment in schizophrenia (SCZ) is ‘functional recovery,’ defined as the attainment of a reasonable degree of normal functioning in work activities, independent living and family and social relationships (Leucht and Lasser, 2006; Jaaskelainen *et al*., 2013). Social dysfunction in SCZ represents one of the major obstacles to reach functional recovery in clinical practice (American Psychiatric Association, 2013; Degnan *et al*, 2018; Javed and Charles, 2018; Gundogmus *et al*., 2021; Ucok *et al*., 2021).

Social withdrawal, defined as a ‘passively or actively reduced social interaction’ (van der Wee *et al.*, 2019), constitutes one of the most observable clinical manifestations of social dysfunction in SCZ. Furthermore, social withdrawal likely represents a causal factor in the development of psychosis in vulnerable subjects (Hoffman, 2007) and has a high impact on the quality of life of SCZ patients (Siegrist *et al.*, 2015; Tremeau *et al*., 2016). During the disease course, delusional thoughts and social distress could lead to a reinforcement of avoidance behaviors, which in turn foster a further worsening of SCZ symptoms (Freeman *et al.*, 2007; Zhong *et al.*, 2017) and increase the risk of hospitalization and clinical relapse (Vazquez Morejon *et al*., 2018). In clinical research, social withdrawal could be a valid and quantifiable real-world indicator of social dysfunction in SCZ (Bilderbeck *et al.*, 2019), but specific and objective assessment tools in clinical practice are still lacking (Buck *et al.*, 2019; van der Wee *et al.*, 2019).

Social dysfunction in SCZ has been associated with a number of sociodemographic, psychopathological and cognitive factors (Kim *et al.*, 2019; Porcelli *et al*., 2020). Among them, neurocognitive deficits are one of the most investigated. Neurocognitive impairment is broadly recognized as a core pathological dimension of SCZ, and various degrees of impairment have been observed in different neuropsychological domains (Heinrichs and Zakzanis, 1998; Keefe *et al.*, 2006). Poor neurocognitive performance has been repeatedly associated with poor functional outcome in SCZ patients (Green, 1996; Lepage *et al.*, 2014). Consistently, in the last decades, cognitive remediation interventions have been developed with the aim to improve general functioning through the enhancement of cognitive skills (Wykes *et al.*, 2011). Nonetheless, knowledge about the most appropriate cognitive targets to improve functional outcomes is still limited (Reeder *et al.*, 2004), contributing to the partial effectiveness of this kind of interventions (McGurk *et al.*, 2007). Past research attempted to investigate whether specific neurocognitive deficits were associated with different aspects of social dysfunction in SCZ. However, most of these studies were based on small and selected samples of patients (Dickinson and Coursey, 2002; McClure *et al.*, 2007) and failed to give consistent results (Green *et al.*, 2000; Evans *et al.*, 2003). Furthermore, outcome measures employed in these studies lacked homogeneity and were mainly related to general domains of functional outcome (e.g. community functioning) (Fett *et al.*, 2011). As a matter of fact, only few studies investigated the association between cognitive functioning and social functioning in large and heterogeneous patient populations (Rosenheck *et al.*, 2006; Bowie *et al.*, 2008; Mohamed *et al.*, 2008; Perlick *et al.*, 2008; Lipkovich *et al.*, 2009; Fervaha *et al.*, 2014). Previous research utilizing the Clinical Antipsychotic Trial of Intervention Effectiveness (CATIE) study data set has analysed different aspects of the correlation between neurocognition and general functioning in SCZ patients. Fervaha and colleagues revealed that neurocognition, together with amotivation, was one of the two significant clinical predictors of 1-year longitudinal functional outcomes [assessed through the Quality of Life Scale (QOLS) (Heinrichs *et al.*, 1984)] (Fervaha *et al.*, 2014). Moreover, Rosenheck and colleagues observed that SCZ patients with a reduced participation in either competitive or noncompetitive employment showed, among clinical features, poorer neurocognitive functioning (Rosenheck *et al.*, 2006). Finally, Mohamed and colleagues found that neurocognition composite score (besides positive and negative symptoms scores) was independently and cross-sectionally correlated with quality of life [assessed through the QOLS (Heinrichs *et al.*, 1984)]. Specifically, the strongest association was observed with the QOLS domain concerning intrapsychic functioning (Mohamed *et al.*, 2008). Nevertheless, none of these studies investigated the association among the different cognitive domains and specific indicators of social dysfunction such as social withdrawal.

Taking into account these considerations, in the present study, we aimed to investigate in a large and heterogeneous sample of SCZ patients cross-sectional associations among different neurocognitive domains and social withdrawal indicator. First, we investigated the associations among neurocognitive domains and social withdrawal indicator in the whole sample and in subsets of patients that significantly differed in terms of cognitive performance. Second, we investigated the sociodemographic and clinical factors associated with social withdrawal. Finally, through a multiple linear regression analyses, we investigated the combined effect on social withdrawal by cognitive domains and additional sociodemographic and clinical factors.

## Methods

### Sample investigated

Data were obtained from CATIE SCZ study (Stroup *et al.*, 2003). CATIE is a multisite randomized controlled trial funded by the National Institute of Mental Health, which aimed to determine the long-term effects and usefulness of antipsychotic medications in persons with SCZ. Details of the study can be found elsewhere (Stroup *et al.*, 2003). For the present study, only the baseline data were considered.

### Measures

Neurocognition was assessed by a battery of tests described in detail in a previous publication (Keefe *et al.*, 2003). The test scores were converted into standardized scores and combined to construct five separate domain scores that measured (a) verbal memory [assessed with the Hopkins Verbal Learning Test (Belkonen, 2011)], (b) vigilance [average of continuous performance test (Cornblatt and Keilp, 1994)], (c) processing speed [shown as the average score for Grooved Pegboard test (Lafayette Instrument Company, 1989), Wechsler Adult Intelligence Scale-R digit symbol test (Wechsler, 1974), and the average of the Controlled Oral Word Association Test (COWAT) (Benton and Hamscher, 1978) and Category Instances (Benton and Hamscher, 1978)], (d) Reasoning [represented by average scores of the Wisconsin Card Sorting Test (WCST) (Kongs *et al.*, 2000) and Wechsler Intelligence Scale for Children-III mazes test (Wechsler, 1991)], and (e) Working Memory [reflecting the average of scores on computerized tests of visuospatial working memory (sign reversed) (Lyons-Warren *et al.*, 2004) and letter-number sequencing (LNS) (Gold *et al.*, 1997)]. These five domains were further combined to obtain a neurocognitive composite score. (see Supplementary Table, Supplemental Digital Content 1, http://links.lww.com/ICP/A96, which illustrates cognitive domains, types of test and measures used).

Social withdrawal was assessed through item 8 (‘Social withdrawal’) of the QOLS (Heinrichs *et al.*, 1984). The QOLS is a 21-item scale based on a semistructured interview that was developed to assess deficit symptoms in SCZ. The scale items range from 0 (highest impairment) to 6 (almost normal) and belong to four different categories: (a) interpersonal relations, (b) instrumental role, (c) intrapsychic foundations, and (d) common objects and activities. The first category (‘Interpersonal relationships’) relates to aspects of interpersonal and social experiences and encompasses items that refer both directly and indirectly to social withdrawal. However, many of them go beyond estimating amount or frequency of social contact to complex and subjective judgements such as the capacity for intimate relationships or the degree of social engagement. For this reason, we decided to use as social withdrawal indicator only the item 8 (‘Social withdrawal’) of this category, which focuses on ‘the degree of which the person actively avoids social interaction due to his/her discomfort or disinterest’ (Heinrichs *et al.*, 1984).

Sociodemographic questionnaires provided information regarding age, ethnicity, sex, marital, employment and educational status (Stroup *et al.*, 2003). SCZ symptoms were assessed through the Positive and Negative Syndrome Scale (PANSS) (Kay *et al.*, 1987).

### Statistical analysis

Bivariate correlations were used to analyze the associations among cognitive domains and social withdrawal indicator first in the whole sample as well as in subsets of patients that were based on the sociodemographic features associated with the neurocognitive composite score (in our case, they were represented by ‘Ethnicity’ and ‘Employment status’) (see Supplementary Table, Supplemental Digital Contents 1 and 2, http://links.lww.com/ICP/A96, which illustrates the correlations of sociodemographic features with neurocognitive performance in all CATIE patients). Analysis of variance (ANOVA) analyses were used to investigate the differences in cognitive performance among groups of patients identified by sociodemographic features. Associations with social withdrawal indicator were investigated by simple regressions and ANOVA, when appropriate. A multiple linear regression analysis was conducted to investigate the variance explained of social withdrawal indicator by continuous and categorical variables. All *P* values were 2-tailed, and statistical significance was set at the standard level of *P* = 0.05. Statistical analyses were conducted using the STATISTICA software package (StatSoft, Inc. Tulsa, Oklahoma, USA).

## Results

Sociodemographic and psychopathological features of the sample are shown in Table 1. For the present study, we considered 761 SCZ patients with available data about social withdrawal and neurocognitive domains.

**Table 1:** Socio-demographic and clinical features of all Clinical Antipsychotic Trial of Intervention Effectiveness patients

Patients (*n*)	761
Ethnicity (%)	Caucasian: 66.36%
	African American: 30.88%
	Others: 2.76%
Male (%)	73.19%
Age (mean ± SD)	40.95 ± 11.04
PANSS total score (mean ± SD)	74.20 ± 17.50
Social withdrawal score (QOLS – Item 8) (mean ± SD)	2.83 ± 1.74
Education (years) (mean ± SD)	12.12 ± 2.21
Marital status (%)	Single: 59%
	Married: 10.91%
	Separated/divorced/widowed: 30.09%
Employment status (%)	Unemployed: 84.83%
	Employed (full time/part time): 15.17%
Verbal memory score (mean ± SD)	0.03 ± 1.00
Vigilance score (mean ± SD)	0.02 ± 1.02
Processing speed score (mean ± SD)	−0.05 ± 1.03
Reasoning score (mean ± SD)	−0.03 ± 1.01
Working memory score (mean ± SD)	−0.01 ± 0.99
Neurocognitive composite score (mean ± SD)	−0.01 ± 1.01

PANSS, Positive and Negative Syndrome Scale; QOLS, Quality of Life Scale.

Regarding the relationship between social withdrawal and cognitive domains, in the whole sample, social withdrawal was associated with a lower score in the neurocognitive composite score (*P* = 0.001; *r* = 0.12) and in three cognitive scale scores: ‘Verbal Memory’ (*P* = 0.0001; *r* = 0.14), ‘Processing Speed’ (*P* = 0.022; *r* = 0.09) and ‘Working Memory’ (*P* = 0.002; *r* = 0.11) (Table 2). Within the latter two domains, only the ‘COWAT and Category Instances test’ (*P* = 0.005) and the ‘LNS Test’ (*P* = 0.0001) resulted associated with social withdrawal.

**Table 2:** Associations among social withdrawal and neurocognitive domains (expressed in *r* values^a^)

Social withdrawal	Verbal memory	Vigilance	Processing speed	Reasoning	Working memory	Neurocomposite score
Whole sample (*n* = 715)	0.14***	0.07	0.09*	0.06	0.11**	0.12**
Caucasian (*n* = 476)	0.17***	0.08	0.13**	0.06	0.11*	0.14**
Non-Caucasian (*n* = 239)	0.09	0.06	0.00	0.06	0.12	0.08
Employed (*n* = 111)	0.16	0.15	0.17	0.28**	0.27**	0.27**
Unemployed (*n* = 602)	0.12**	0.04	0.05	0.01	0.07	0.07

a *r* value indicates that greater social withdrawal is associated with poorer neurocognitive performance, due to the method of scoring social withdrawal (see main text).

* *P* < 0.05;

** *P* < 0.01;

*** *P* < 0.001.

Concerning the relationship between social withdrawal and cognitive domains in the two subsets of patients with better cognitive performance (‘Caucasian’ and ‘Employed’) (see Supplementary Table, Supplemental Digital Contents 1 and 2, http://links.lww.com/ICP/A96, which illustrates the correlations of socio-demographic features with neurocognitive performance in all CATIE patients), social withdrawal was associated with a lower score in the neurocognitive composite score (*P* = 0.002 and *P* = 0.005, respectively). In the ‘Caucasian’ subset, the associations found in the whole sample between neurocognitive domains scores and social withdrawal were confirmed [‘Verbal Memory’ (*P* = 0.0001), ‘Processing Speed’ (*P* = 0.006) and ‘Working Memory’ (*P* = 0.014)]. On the other hand, in the ‘Employed’ subset, higher levels of social withdrawal were associated with poorer ‘Reasoning’ (*P* = 0.003) and ‘Working Memory’ (*P* = 0.004) scores. Within the subsets of patients with lower cognitive performance, no association was found in the ‘Non-Caucasian’ group between social withdrawal and neurocognitive scores, whereas in the ‘Unemployed’ group, social withdrawal was associated only with ‘Verbal Memory’ score (*P* = 0.003).

Regarding the relationships between social withdrawal and the sociodemographic and clinical features, social withdrawal was associated with lower education (*P* = 0.026), employment status (‘Unemployed’ patients showed higher social withdrawal) (*P* = 0.002) and higher PANSS positive symptoms score (*P* = 0.0001) (see Supplementary Table, Supplemental Digital Contents 1 and 3, http://links.lww.com/ICP/A96, which illustrates the correlations of sociodemographic and clinical features with social withdrawal in all CATIE patients). PANSS negative and general psychopathological symptoms’ scores were associated with social withdrawal as well (both *P* < 0.001), although this relationship was likely influenced by the overlap of specific items (e.g. N4 = ‘Passive/apathetic social withdrawal’; G16 = ‘Active social avoidance’) with social withdrawal indicator. No association was found among age, sex, ethnicity, marital status and social withdrawal indicator.

In the multiple regression analysis, we excluded PANSS negative and general psychopathological symptoms’ scores due to the presence of items (e.g. N4 = ‘Passive/apathetic social withdrawal’; G16 = ‘Active social avoidance’) that overlap with social withdrawal indicator. Considering together cognitive domains (‘Verbal Memory,’ ‘Processing Speed’ and ‘Working Memory’) and additional clinical and sociodemographic factors (education years, PANSS positive symptoms score and employment status), the model explained the 8% of the total variance of social withdrawal indicator (F = 8.69; *P* = 0.0001). Among all the factors, only PANSS positive symptoms (*P* < 0.0001) and ‘Verbal Memory’ (*P* = 0.033) scores were still associated with social withdrawal.

## Discussion

The aim of our study was to investigate cross-sectional associations among different neurocognitive domains and social withdrawal in a large and heterogeneous sample of SCZ patients. Furthermore, we investigated the variance explained of social withdrawal indicator by cognitive domains and additional sociodemographic and clinical factors.

First, we found a negative association between global cognitive functioning and the degree of social withdrawal (Table 2). Our result is consistent with previously published results, although the association appears slightly weaker (*r* = 0.12) in comparison with the result of a recent review reporting a medium-sized average association (*r* = 0.14–0.26) between overall neurocognition and functional outcome in SCZ (Halverson *et al.*, 2019). Considering the high percentage of variation (up to 70%) of effect sizes of this association due to heterogeneity among studies, our findings can be partly explained by the choice of a social functioning measure that focused on a particular aspect of social behavior (i.e. social withdrawal) in comparison with more general measures of functional outcome considered in the review’s studies (such as community functioning, social behavior in the milieu, etc.) (Fett *et al.*, 2011).

Second, our results are consistent with previous literature in confirming the associations among neurocognitive performance and different measures of social functioning in SCZ patients (Bowie *et al.*, 2008; Perlick *et al.*, 2008; Lipkovich *et al.*, 2009). Indeed, previous studies showed that discrete neuropsychological domains are associated in different and specific ways with various indicators of social functioning. In particular, our findings suggested the association of three specific cognitive domains (i.e. verbal memory, processing speed and working memory) with social withdrawal behavior. Among them, ‘verbal memory’ scores were most strongly associated with social withdrawal indicator (*r* = 0.14). In SCZ, verbal memory deficits appear to be independent of clinical state or medication effects (Bilder *et al.*, 2000; Goldberg *et al.*, 2007) and are considered by some authors as the greatest cognitive predictive factors of social outcome (Green *et al.*, 2000; Toulopoulouand and Murray, 2004). Consistently, in previous studies, verbal memory deficits have been associated with impairments in recreational activities (Milev *et al.*, 2005), interpersonal relationships (Ueoka *et al.*, 2011) and social problem solving (Green, 1996).

On the other hand, ‘processing speed’ and ‘working memory’ scores were associated with social withdrawal indicator as well (*r* = 0.09 and *r* = 0.11, respectively). Within these two domains, the tasks associated with social withdrawal assessed, respectively, verbal fluency skills (assessed by COWAT and Category Instances test) and auditory working memory skills (evaluated by LNS test). Processing speed and working memory deficits are thought to contribute to impairments in other cognitive domains in SCZ (Silver *et al.*, 2003; Rodriguez-Sanchez *et al*., 2007), and their association with general functioning is well-established in SCZ, as well as their effects on specific functional domains (e.g. social behavior, community functioning and competitive employment) (Dickinson and Coursey, 2002; Kurtz, 2006).

In a previous large study focused on the specific relationships between neurocognition and general functioning in SCZ (Bowie *et al.*, 2008), verbal memory was found to predict only functional competence (an indicator of everyday functioning skills), whereas processing speed and working memory were associated with both functional and social competence and performance domains (with interpersonal behavior and work performance, respectively) (Bowie *et al.*, 2008). In another study with a large sample of SCZ patients, Perlick and colleagues (Perlick *et al.*, 2008) showed an association among different cognitive domains and functional status assessed through QOLS (Heinrichs *et al.*, 1984). Only motor and memory skills were associated with social behavior (evaluated by the ‘Interpersonal relationship’ domain of QOLS). Finally, Lipkovich and colleagues (Lipkovich *et al.*, 2009) found in a sample of 414 SCZ outpatients that multiple functional domains [assessed with QOLS (Heinrichs *et al.*, 1984)] were cross-sectionally associated with processing speed, working memory and verbal memory, although only processing speed skills were associated with the ‘Interpersonal relationship’ domain of QOLS. These results consistently suggested specific associations among verbal memory, processing speed and working memory with different aspects of general functioning and social behavior, as showed by our results. Nonetheless, the paucity of available studies prevents us from drawing clear conclusions about the specificity and the reproducibility of these associations.

Exploratory analyses found in ‘Caucasian’ and ‘Employed’ subgroups associations between the neurocognitive composite score and social withdrawal score, but the same association was not found in ‘Non-Caucasian’ and ‘Unemployed’ subgroups. Moreover, in the ‘Employed’ subgroup, we found an association between social withdrawal and ‘Reasoning’ domain. Therefore, it seems that the relationship between social withdrawal and neurocognition tends to be mainly expressed within the ‘Caucasian’ and ‘Employed’ subgroups. Interestingly, although the ‘Unemployed’ subgroup results to be strongly associated with social withdrawal, we found only one correlation between social withdrawal and cognitive domains (specifically with ‘Verbal Memory’ domain) within this subgroup. We assume that ‘Verbal Memory’ might have a critical impact on social withdrawal, and this is somehow confirmed by the persistence of a significant association between this cognitive domain and social withdrawal in the multiple regression analysis. Although potentially interesting, these results should be interpreted with care because of the intrinsic increased risk of false-positive findings in exploratory analyses and of the limited characterization of the different ethnicities within the sample.

Regarding sociodemographic features, social withdrawal was associated with lower education and ‘Employment’ status (with ‘Unemployed’ patients showing higher degree of social withdrawal). These results are consistent with the previous literature. Indeed, several studies showed that low intelligence quotient (IQ) and scholastic underperformance represent predictive factors of further worse functional outcome (Barnett *et al.*, 2006; Leeson *et al.*, 2009). Conversely, the presence of a lower IQ and cognitive impairment after SCZ onset was found to be predicted by poor premorbid social functioning (Stefanatou *et al.*, 2018). The higher degree of social withdrawal shown by unemployed patients can be explained in a bidirectional way as well. It could depend either on the negative effect of preexisting social deficits on the attainment of employment by SCZ patients (Himle *et al.*, 2014) or on financial constraints due to unemployment that would impede the access to recreational activities (Porcelli *et al.*, 2020).

Concerning psychopathological symptoms, social withdrawal was significantly associated with each of the PANSS subscale scores (positive, negative and general psychopathologies). Our finding is consistent with previous studies, which reported stronger associations with social dysfunction for negative and general symptoms (Rosenheck *et al.*, 2006; Mohamed *et al.*, 2008; Savilla *et al.*, 2008; Kim *et al.*, 2019). On the other hand, the observed relationship among negative and general psychopathology scale scores with social withdrawal was quite predictable because of the contiguity and the overlap of several items of the two rating scales with social withdrawal indicator (e.g. N4 = ‘Passive/apathetic social withdrawal’; G16 = ‘Active social avoidance’). In addition, numerous other items concerning depressive or anxious states and cognitive alterations (e.g. G10 = ‘Disorientation’ and G11 = ‘Poor attention’) may contributed to strengthening this correlation (Kay *et al.*, 1987).

Finally, the multiple regression analysis reported a total explained variance (calculated considering cognitive, clinical and sociodemographic factors) of the social withdrawal indicator of 8%. Our findings showed a smaller explained variance of social functioning by cognitive, clinical and sociodemographic factors in comparison with previously reported findings (Savilla *et al.*, 2008; Yamauchi *et al.*, 2008). Nonetheless, this could be due to the exclusion in our analyses of PANSS negative and general psychopathological scores (due to the presence of items that overlap with the social withdrawal indicator, as previously stated) normally considered in past studies and by the choice of a specific measure of social dysfunction (represented by social withdrawal) that could have further reduced the strength of the association.

### Limitations of the study

There are several limitations in this study that need to be acknowledged. First, the study was based on cross-sectional data not allowing causal inferences that require longitudinal observations. Second, the use of a post hoc and derived measure of social withdrawal (item 8 of QOLS) can be arguable because it lacks specificity and direct validation for the purpose. Moreover, single-item indices generally have more measurement errors than multi-item indices reducing the magnitude of potential correlations with other measurements. Furthermore, the evaluation of social withdrawal was based on clinical judgement [QOLS (Heinrichs *et al.*, 1984)] that could be affected by bias due to its subjective nature. At this moment in time, efforts are ongoing to develop and implement new longitudinal, quantitative and objective measures of social functioning using digital phenotyping methods (Jongs *et al.*, 2020), and future studies are needed to understand the relationship between subjective and objective measures for this behavioral domain. Finally, since we decided not to apply any statistical correction because our analyses were all hypothesis-driven, we set the statistical significance at the standard level of *P* < 0.05 with the increased risk of false positive findings, particularly for the exploratory analyses.

## Conclusion

Our study showed that in a large and real-world representative sample of SCZ patients, social withdrawal was associated with neurocognitive deficits involving verbal memory, processing speed and working memory domains. Our results confirmed the wide heterogeneity and specificity of the correlation between neurocognitive domains and indicators of functional outcome in SCZ (Bowie *et al.*, 2008). Furthermore, they underlined the importance of certain neurocognitive abilities compared with others with regard to their correlation with an essential measure of social functioning such as social withdrawal.

For future research, our findings suggest the need for an increasingly comprehensive analysis of the neuropsychological factors related to the different aspects of social dysfunction and for a greater availability of homogeneous and objective evaluation tools (both regarding neurocognitive skills and social functioning measures) in order to extend our knowledge about the precise correlates of disability and the potential treatment targets in SCZ.

## Acknowledgements

The authors would like to thank the NIMH for providing access to data on CATIE sample. The authors would also like to thank the authors of previous publications in this dataset and, foremost, the patients and their families who agreed to be enrolled in the study. Data were obtained from the limited access datasets distributed from the NIH-supported ‘Clinical Antipsychotic Trials of Intervention Effectiveness’ (NIMH Contract No. N01MH900001). The PRISM project (www.prism-project.eu) leading to this application has received funding from the Innovative Medicines Initiative 2 Joint Undertaking under grant agreement No 115916. This Joint Undertaking receives support from the European Union’s Horizon 2020 research and innovation programme and EFPIA. D.D.D. wrote the manuscript draft, performed statistical analyses, managed the formatting and submission. S.P. performed statistical analyses and extensively revised the manuscript draft. D.D.R., E.M.P., M.J.K., and A.B. reviewed the manuscript. A.S. designed the study and reviewed the manuscript.

Participants gave written informed consent to participate in protocols approved by local institutional review boards.

The datasets used and/or analyzed during the current study are available from the corresponding author on reasonable request.

This publication reflects only the authors’ views neither IMI JU nor EFPIA nor the European Commission are liable for any use that may be made of the information contained therein.

### Conflicts of interest

A.S. is or has been a consultant to or has received honoraria or grants unrelated to the present work from: Abbott, Abbvie, Angelini, Astra Zeneca, Clinical Data, Boheringer, Bristol Myers Squibb, Eli Lilly, GlaxoSmithKline, Innovapharma, Italfarmaco, Janssen, Lundbeck, Naurex, Pfizer, Polifarma, Sanofi, and Servier. For the remaining authors, there are no conflicts of interest.

## Supplementary Material


