# Supplementary material for: Social withdrawal and neurocognitive correlates in schizophrenia
Source: Int Clin Psychopharmacol. 2022 Jan 31;37(3):102–9. doi: 10.1097/YIC.0000000000000395 (PMC8969845; doi:10.1097/YIC.0000000000000395)
Supplement: Supplementary file 1 [file icp-37-102-s001.pdf]

Supplemental Digital Content 1. Table that illustrates cognitive domains, types of test and measures used

Cognitive domain	Type of test	Description of test and measure used
Verbal Memory	-Hopkins Verbal Learning Test {Belkonen, 2011 #91}	12 nouns read aloud for three consecutive trials<br>*Measure*: number of items recalled on each trial
Vigilance	-Continuous Performance Test {Cornblatt, 1994 #92}	Reaction to the presentation of numbers on a computer screen with three 150-trial conditions of increasing difficulty<br>*Measure*: mean reaction sensitivity
Processing Speed	-Grooved Pegboard test {Lafayette, 1989 #93}	Insert in a particular order 25 pegs into randomly positioned slots in two 45-second trials<br>*Measure*: mean number of pegs inserted
	-WAIS-R digit symbol test {Wechsler, 1974 #94}	Each number is associated with a form. Copy as many forms associated with the number as possible in 90 seconds<br>*Measure*: mean of forms accurately copied
	-COWAT {Benton AL, 1978 #95}	Create as many words as possible that start with a given letter (F, A or S) in three 60-second trials<br>*Measure*: mean number of words
	-Category Instances {Benton AL, 1978 #95}	Generate as many words as possible within categories of animals, fruits and vegetables in 60 seconds<br>*Measure*: mean number of words
Reasoning	-WCST-64P {Kongs SK, 2000 #97}	Complex task of abstraction, set-shifting and problem solving.<br>*Measure*: number of preservative errors and completed number of categories
	-WISC-III mazes {Wechsler, 1991 #98}	Use a pencil to try to draw through a series of nine mazes without entering blind alleys<br>*Measure*: raw score
Working Memory	-Computerized test of visuospatial working memory {Lyons-Warren, 2004 #99}	Focus on a central fixation cross on a computer screen while a cue appears at random location on the screen. Watch geometric shapes appear and then press the spacebar when diamond shape appears. After a delay, when the cross reappears point to where cue appeared at the beginning. There are three conditions (no delay, 5-s delay and 15-s delay) and eight trials of each condition<br>*Measure*: mean error for each type of trial
	-Letter-number sequencing {Gold, 1997 #100}	Clusters of letters combined with numbers are aurally presented to the patient which is asked to reorder the cluster and tell the experimenter the numbers first, from lowest to highest, then the letters in alphabetical order<br>*Measure*: number of correct sequences.

WAIS-R= Wechsler Adult Intelligence Scale test-revised; COWAT= Controlled Oral Word Association Test; WCST-64P= Wisconsin card sorting test, 64-card computerized version; WISC-III= Wechsler Intelligence Scale for Children third edition

Supplemental Digital Content 2. Table that illustrates the correlations of socio-demographic features with neurocognitive performance in all CATIE patients

	**Neurocognitive Composite Score (NCS)[a]**	**Effect**
**Sex**	F=0.060<br>p=0.807	
**Ethnicity**	F=12.357<br>**p=0.0005**	**Caucasian = ↑ NCS[a]**
**Marital status**	F=1.894<br>p=0.110	
**Employment status**	F=7.941<br>**p=0.0004**	**Employed = ↑ NCS[a]**

[a]calculated by averaging the standardized scores (z-scores) of the summary scores of the five neurocognitive domains, as described in the text

Supplemental Digital Content 3. Table that illustrates the correlations of socio-demographic and clinical features with social withdrawal in all CATIE patients

	**Social Withdrawal Score (SWS)[a]**	**Effect**
**Age**	r=-0.048<br>p=0.189	
**Sex**	F=0.000<br>p=1.000	
**Ethnicity**	F=0.116<br>p=0.732	
**Education (yrs)**	r= 0.081<br>**p=0.026**	**↑ Education = ↑ SWS[a]**
**PANSS[b] (positive symptoms score)**	r=-0.233<br>**p=0.0001**	**↑ PANNS[b] = ↓ SWS[a]**
**PANSS[b] (negative symptoms score)**	r=-0.355<br>**p=0.001**	**↑ PANNS[b] = ↓ SWS[a]**
**PANSS[b] (general psychopathological symptoms score)**	r=-0.320<br>**p=0.001**	**↑ PANNS[b] = ↓ SWS[a]**
**PANSS[b] (total score)**	r=-0.372<br>**p=0.001**	**↑ PANNS[b] = ↓ SWS[a]**
**Marital status**	F=1.755<br>p=0.135	
**Employment status**	F=6.487<br>**p=0.002**	**Unemployed = ↓ SWS[a]**

[a]assessed through the item 8 (“Social withdrawal”) of the Quality of Life Scale (QOLS); lower scores in this measure are indicative of higher social withdrawal, as described in the text

[b]PANSS= Positive And Negative Syndrome Scale
